# Reducing user fees for primary health care in Kenya: Policy on paper or policy in practice?

**DOI:** 10.1186/1475-9276-8-15

**Published:** 2009-05-08

**Authors:** Jane Chuma, Janet Musimbi, Vincent Okungu, Catherine Goodman, Catherine Molyneux

**Affiliations:** 1 KEMRI/Wellcome Trust Programme, PO Box 230, Kilifi, Kenya; 2 Health Policy Unit, London School of Hygiene & Tropical Medicine, Keppel St, London, WC1E 7HT, UK; 3 Centre for Tropical Medicine, University of Oxford, Oxford, UK

## Abstract

**Background:**

Removing user fees in primary health care services is one of the most critical policy issues being considered in Africa. User fees were introduced in many African countries during the 1980s and their impacts are well documented. Concerns regarding the negative impacts of user fees have led to a recent shift in health financing debates in Africa. Kenya is one of the countries that have implemented a user fees reduction policy. Like in many other settings, the new policy was evaluated less that one year after implementation, the period when expected positive impacts are likely to be highest. This early evaluation showed that the policy was widely implemented, that levels of utilization increased and that it was popular among patients. Whether or not the positive impacts of user fees removal policies are sustained has hardly been explored. We conducted this study to document the extent to which primary health care facilities in Kenya continue to adhere to a 'new' charging policy 3 years after its implementation.

**Methods:**

Data were collected in two districts (Kwale and Makueni). Multiple methods of data collection were applied including a cross-sectional survey (n = 184 households Kwale; 141 Makueni), Focus Group Discussions (n = 12) and patient exit interviews (n = 175 Kwale; 184 Makueni).

**Results:**

Approximately one third of the survey respondents could not correctly state the recommended charges for dispensaries, while half did not know what the official charges for health centres were. Adherence to the policy was poor in both districts, but facilities in Makueni were more likely to adhere than those in Kwale. Only 4 facilities in Kwale adhered to the policy compared to 10 in Makueni. Drug shortage, declining revenue, poor policy design and implementation processes were the main reasons given for poor adherence to the policy.

**Conclusion:**

We conclude that reducing user fees in primary health care in Kenya is a policy on paper that is yet to be implemented fully. We recommend that caution be taken when deciding on how to reduce or abolish user fees and that all potential consequences are carefully considered.

## Introduction

Removing user fees in primary health care services is one of the most critical policy issues being considered in Africa. User fees were introduced in many African countries during the 1980s as a response to the significant economic constraints and increasing donor pressure. Proponents of user fees argued that fees would generate additional revenue, which could be used to improve equity and efficiency; that graduated fees would encourage use of low cost primary health care services rather than expensive referral facilities; and that they would improve targeting of resources by reducing unnecessary demand [1]. More than two decades since their introduction, evidence shows that user fees have done more harm than good. They impact negatively on the demand for health care, contribute towards household poverty, promote inequities and generate little revenue [2-9]. Waivers have generally failed to protect the poor: deciding who should benefit is difficult; patients have little knowledge of waiving mechanisms; and the process of acquiring a waiver is complex and time consuming for both patients and providers [10].

Concerns regarding the consequences of user fees and other Out-of Pocket payments (OOPs) have led to a recent shift in health financing debates in Africa, away from user fees to mechanisms that encourage prepayment and tax funding [11]. Uganda and South Africa have already eliminated user fees in all primary health care facilities. Kenya replaced variable user fees for primary health care with a minimum flat rate, while Zambia has eliminated user fees in all rural government facilities. Other countries including Tanzania, Burundi and Burkina Faso are considering-or are in the process of-user fees removal [12].

Recent experiences with user fees removal in Africa have generally led to increased utilisation of health care services. In Uganda, almost all health services immediately reported a 50–100% increase in attendance, with about 50% of the increase being reported among adults in the poorest quintile [13]. Similar patterns were reported in South Africa, although the initial increases were not always maintained over time, and increased utilisation of curative services appeared to crowd out preventive care and demoralised staff [14,15].

Initial increases in utilisation have in some cases been used to justify fee removal. However, evaluations of user fees removal have mainly been conducted within the first year of policy implementation, the period when the expected positive impacts are likely to be highest [13,16]. Immediate evaluation of user fees removal can overestimate policy success and may fail to capture the experiences of policy implementers in their attempts to adhere to new policies. Whether or not increased utilisation rates are sustained, the extent to which facilities continue to adhere to 'new' policies, and the impact on service provision and quality of care have hardly been explored. We conducted this study to document the extent to which primary health care facilities in Kenya continue to adhere to a 'new' charging policy 3 years after its implementation. We also gathered providers' opinions on the new policy, communities' understanding of the recommended charges under the new policy, and their perceived impact on service quality.

### An overview of health care financing in Kenya

Like many African countries, Kenya introduced user fees in public health facilities in the 1980s, reflecting a combination of factors including poor economic performance, inadequate financial resources, declining budget allocations and international donor pressure [6]. Following initial implementation in 1989, user fees were suspended in 1990 and reintroduced in 1991 [5,10]. Facilities set user fees locally with the support of Health Facility Committees (HFCs). Fees were charged for individual services like drugs, injections, and laboratory services. Revenue collected was returned to the district level and facilities developed detailed plans for spending 75% of the revenue. A waiving policy to protect the poor was put in place, and children below five years were exempted from all charges, but in reality waiving and exemption mechanisms hardly existed [6].

Evidence on user fees and other OOPs in Kenya revealed that health care charges were a significant barrier to access especially among the poorest populations [5,10,17]. To address these equity concerns and partly to fulfill a political pledge, the Minister for Health announced that from 1^st ^July 2004, services at dispensaries and health centres would be free for all citizens, except for a minimum registration fee of Kenya Shillings (KES) 10 and KES 20 respectively (approximately 2008 USD 0.2 and 0.3 respectively). Under the new policy – commonly referred to as the 10/20 policy – children aged below five years and specific health conditions such as Malaria and Tuberculosis are exempted from payment. Registration fees should also be waived for the poor. In a further development, all fees for deliveries in public health facilities were abolished in July 2007.

An evaluation of the 10/20 policy was conducted in 2005 [16]. Using data collected in 2004, the evaluation reported high adherence to the new policy and an increase in utilisation of 70%. This increase was not sustained, although utilisation remained 30% higher than prior to the 10/20 policy. The policy was also reported to be popular among patients, but not with health workers.

Another related intervention was a pilot project introduced in Coast province in late 2005 that provided funds to compensate facilities for lost user fees revenue. Direct Facility Funding (DFF) was supported by the Danish International Development Agency (DANIDA) and was meant to enable facilities to adhere to the 10/20 policy. Facilities receiving DFF were required to open a bank account where funds were transferred directly from the national level. Decisions on how to spend DFF funds were made at the facility level, although facilities were expected to comply with some guidelines. For example, a maximum of 30% of the individual facility funds could be spent on allowances, but the funds could not be used to purchase drugs or laboratory services. Drugs and equipments are supplied by the Kenya Medical Suppliers Agency (KEMSA). Plans are under way to scale up DFF country wide, with the first round of funds likely to be paid in 2009.

The 10/20 policy and DFF are potentially good developments for the Kenyan health sector. It is therefore important to document the extent to which facilities in areas with and without DFF continue to adhere to the 10/20 policy a few years after implementation, the contribution of this policy towards improving access to health care, and how both health workers and community members perceive the policy.

## Methods

### Study area

Data were collected in two districts: Kwale in Coast province and Makueni in Eastern province. The two districts are semi-arid with high poverty levels. Agriculture is the main source of income in both districts, although Kwale has a significant tourism sector. The districts were chosen purposively from a wider study conducted in four districts in Kenya. The aim of the wider study was to explore barriers of access to malaria control interventions among the poorest population. Reducing user fees for primary health care and providing free malaria treatment in all government facilities in Kenya were some of the interventions considered in the study as having the potential to promote access to health care among the poor, particularly because malaria is the major cause of mortality and morbidity in both districts. Kwale district was selected because it is located in Coast province where the DFF was being piloted, while Makueni was chosen because there was no DFF in the district thus acting as a 'control' site, and it was the closest to Kwale of the remaining three districts. Both districts were also the closest to the researchers' institutional base.

### Data collection and analysis

Data collection took place in May and June 2007. Both qualitative and quantitative data collection methods were applied including interviews with health workers and HFC members, exit interviews with patients, Focus Group Discussions (FGDs) and a cross-sectional survey. A multi-stage sampling approach was conducted to select survey households for the wider study. The same households responded to questions regarding user fees reduction:

• First, locations (the 2^nd ^lowest administrative unit in Kenya) were selected using poverty indicator maps [18]. The maps classified locations within each district into poverty quintiles based on the percentage of the population living below the international poverty line of USD 1 per day. We identified all locations which fell within the two poorest quintiles in each district. We assumed that by focusing on the two poorest quintiles we stood a high chance of reaching the poorest households that are expected to have poor access to health care.

• The locations in the two poorest quintiles contained a total of 73 Enumeration Areas (EAs) (28 Kwale; 45 Makueni). We randomly selected 4 EAs per district.

• We updated the homestead list and randomly selected 100 homesteads per district from participating EAs.

• All households in the homestead were included in the study (n = 184 Kwale; 141 Makueni).

Key information gathered through the household survey included community members' understanding of recommended charging levels at the dispensaries and health centres, their understanding of waivers and exemptions and their experiences in securing waivers.

For the facility-level data collection, we selected 14 health facilities in Kwale (10 dispensaries and 4 health centres) and 20 facilities in Makueni (13 dispensaries and 7 health centres). These facilities were sampled purposively because they were located close to the areas where the household survey took place and were therefore likely to serve the community under study. Semi-structured interviews were conducted with facility in-charges to enable a detailed inquiry into the factors surrounding policy change and service provision. Key questions covered in the semi-structured interviews were: charging levels for adults, under fives and specific illness conditions; whether or not they adhered to the policy and factors facilitating/hindering adherence; processes for granting waivers; and perceptions on 10/20 policy and it impacts on service provision.

Exit interviews were conducted with patients (or guardians in the case of children) seeking treatment on the date of the interview (n = 175 Kwale; 184 Makueni). All participants attending the facility between 8.30 am to 1.00 pm on the date of interview were approached to take part in the study. Only 8 people declined. Exit interviews captured data on the type of services received, amount paid and perceptions of quality of care. These data were supplemented by 12 FGDs, selected on the basis of gender and place of residence.

Quantitative data were double entered into Fox-Pro version 9.0 and later transferred to STATA (9.0) for analysis. Data from the FGDs were tape-recorded, transcribed and typed into Ms-Word, while notes were taken for semi-structured interviews. Qualitative data were analyzed manually using content analysis to identify common themes and sub-themes. Key themes were organised around the main topics of interest, for example, charging levels and charging strategies, adherence to the policy, impacts of policy on service provision (positive and negative) and community understanding of the policy.

## Results

### Description of study participants and health facilities

Dispensary in-charges were mainly nurses while all health centres were headed by a clinical officer (Table 1). Most of the in-charges had worked at the participating health facility for more than three years. The median number of health workers per facility was 2 (range = 1–24). The range of services provided differed across facilities, with some offering laboratory services (n = 5 Kwale; 11 Makueni), inpatient care (n = 2 Kwale; 11 Makueni) and delivery services (n = 6 Kwale; 19 Makueni). Exit interview participants had mainly visited the facility for curative services (81% Kwale; 61% Makueni) and immunisations for children below five years (12% Kwale; 21% Makueni). Participants from Makueni were generally better educated than those from Kwale. Only 42.9% of the respondents had some years of formal education in Kwale compared to 80.0% in Makueni. Household survey respondents were mainly household heads (59.2% Kwale; 50.4% Makueni) or their spouses (33.7% Kwale; 39.0% Makueni).

**Table 1:** Basic characteristics of health workers and description of services offered at participating facilities

	**Kwale**		**Makueni**	
	Dispensaries (n = 10)	Health centres (n = 4)	Dispensaries (n = 13)	Health Centres (n = 7)

In charges				
• Clinical officers	1	4	0	7
• Nurses	9	0	13	0
• Public Health Officers	0	0	0	0

All staffs				
• Clinical officers	2	8	0	8
• Nurses	18	19	23	37
• Public Health officers	9	3	0	12

Services				
• Laboratory	2	3	4	7
• Inpatient	0	2	0	6
• Deliveries	3	3	12	7
• Voluntary Counseling and Testing (VCT)	0	2	0	2

### Communities' understanding of recommended charges

Communities had very limited understanding of the recommended charges under the 10/20 policy. About a third of all household survey respondents (regardless of whether they visited dispensaries and health centres) could not correctly state the recommended charges for dispensaries, while half reported that they did not know the official charges for health centres (Table 2). Only 26.1% of respondents in Kwale and 41.1% in Makueni reported that children under five should be exempted from payments, while a further 25.0% in Kwale and 7.1% in Makueni reported that the poor are eligible for free treatment (Table 3).

**Table 2:** Respondents' perceptions of government guidelines on user fees for all households that participated in the cross-sectional survey

	**Dispensaries**		**Health Centres**	
Amount	Kwale n = 184 (%)	Makueni n = 141 (%)	Kwale n = 184 (%)	Makueni n = 141 (%)

KES 10	55 (28.9)	25 (17.7)	1 (0.5)	2 91.4)

KES 20	25 (13.6)	36 (25.5)	13 (7.1)	20 (14.2)

KES 50	4 (2.2)	4 (2.8)	25 (13.6)	9 (6.4)

Depends on drugs	51 (27.7)	33 (23.4)	57 (30.1)	46 (32.6)

Do not know	39 (21.2)	39 (27.7)	87 (47.3)	61 (43.3)

Other	10 (5.4)	4 (2.8)	1 90.5)	3 (2.1)

**Table 3:** Household survey respondents' perceptions of government guidelines on groups who should receive free treatment (% in brackets)

	Kwale n = 184 (%)	Makueni n = 141 (%)
Under 5s	48 (26.1)	58 (41.1)

The poor	46 (25.0)	10 (7.1)

Elderly	17 (9.2)	1 (0.7)

Pregnant women	11 (6.0)	5 (3.5)

Do not know	95 (51.6)	73 (51.8)

Other	4 (2.2)	2 (1.4)

*Total adds up to >100% because some respondents reported more than one category (N = 37 Kwale; 8 Makueni)

Results from FGDs supported the household survey findings. Charging levels were said to differ between facilities, with most charging higher than the recommended fees. There was confusion regarding the existing charging levels however, with people from the same community reporting different levels at their local facility. People reported being charged for various items including drugs, injections and cards:

"For children the charges are KES 10 for tablets and KES 20 for each injection. Adults pay KES 10 for registration. The other payment depends on the illness and could range from KES 150 to KES 250." (FGD, Women)

On a few occasions, participants demonstrated some awareness of what the recommended charges were, but it was not always clear what the charges were for. They highlighted the need for clarity and communication from the authorities regarding charging levels:

"You pay KES 20 for the card then you are told to pay for drugs. If you need an injection you are told to pay another KES 50...you see I get confused. Did the government not say that we should only pay for the card [meaning registration]?" (FGD, Men)

"There are some things that should be paid for and there are others that do not require payment [under the 10/20]. But you find that where money is required, you are asked to pay and where payment is not required, you are also asked to pay. Now the government needs to tell us what we should pay for and what is provided for free." (FGD, Women)

### How much are facilities charging?

Facility-level data showed that reported levels of charges differed between and within districts, with very few facilities reporting strict adherence to the 10/20 policy. Adherence to the 10/20 policy in this paper refers to a situation where dispensaries and health centers charged registration fees of KES 10 and KES 20 respectively, with children under the age of five and some illness conditions (e.g. malaria and tuberculosis) being exempted from paying the registration fees. Any extra charges (e.g. laboratory fees, cards, drugs, delivery) indicate non-adherence to the policy. Facilities were classified as adherent/non-adherent to the policy based on reports on these charges from health workers interviews. The findings presented therefore indicate levels of reported adherence, which might be different from verified adherence.

Facilities in Makueni were more likely to adhere to the 10/20 policy than those in Kwale. Only 4 facilities in Kwale adhered to the policy compared to 10 in Makueni. Facilities charged for different kinds of services including: registration, injections, drugs, deliveries and laboratory services. Patterns of non-adherence differed between districts. For example, all facilities that did not comply with the 10/20 in Kwale charged registration fees to under fives (n = 10), while 4 dispensaries increased the registration fees from KES 10 to KES 20 and 3 health centres from KES 20 to KES 40. In contrast, only two facilities in Makueni charged registration fees to children under five and none reported increasing the registration fees.

Results from exit interviews supported findings from health worker interviews. About 57% of exit interview participants in Kwale and 20% in Makueni who reported being charged for treatment on the date of the interview paid more than the recommended charges under the 10/20 policy. Median levels in Makueni reflected the recommended charges under the 10/20 policy, while median charges in Kwale were double the official amount (Table 4).

**Table 4:** Mean and median charges for exit interview participants who reported paying money for treatment/services on the date of interview

	**Dispensaries**		**Health Centres**	
Amount	Kwale n = 98	Makueni n = 73	Kwale n = 14	Makueni n = 32

Mean	25	14	52.3	36

Median	20	10	40	20

Range	100–220	10–100	20–230	20–100

### Factors influencing adherence to the 10/20 policy

Drug shortage was the most common reason given for poor adherence to the policy. Drug shortages were common during the peak illness seasons and towards the end of the drug supply period (i.e. at the end of every quarter before facilities received their quarterly supply). A review of the drugs in stock indicated that most facilities in Kwale (n = 10) and 2 in Makueni lacked at least one essential drug on the date of the interview. To cope with drug shortages, facilities either increased the registration fees or decided to charge extra fees for the drugs:

"The dispensary committee decided to increase the charges because the facility often experiences drug shortages. They buy drugs and sell them to the patients at KES 50 for each type of drug." (Interview, facility in-charge, Makueni)

In some cases, concerns were not about an overall lack of drugs, but a failure to ensure that the content of the drug kits suited local needs. For example, it was reported that typhoid was a common health problem in Kwale but the government drug kit did not contain typhoid drugs. Consequently facilities purchased drugs that were not provided by the government and issued them to patients at an extra fee.

Declining revenue also prevented facilities from adhering to the 10/20 policy. It was reported that the recommended registration fees were too low, were often insufficient to meet the running costs of the facility, and that budgetary allocations from the government were inadequate. Adjusting fees was reported as an important coping strategy to ensure that service provision was maintained:

"They increased registration fees to KES 20 per person including children following discussions with the community because it became impossible to continue providing services under the 10/20 policy." (Interview, facility in-charge, Kwale)

"They always experience cash shortages because what the facility budgets for is not what they receive from the government. With the new charges [10/20], utilisation of services is high and available drugs can not meet the high demand...so they cope by increasing the charges" (Interview, facility in-charge, Kwale)

Other reasons given were to raise funds to pay support staff and to meet the costs of laboratory services which mainly operated on a private basis.

Dispensaries were more likely to adhere to the 10/20 policy than health centres. Out of the health centres included in the study only one charged the recommended fees. The main reason health centres failed to comply is that they offered a wide range of services including inpatient care. Some health centres were large and had structures similar to sub-district hospitals but were still required to operate under the 10/20 policy:

"The facility is classified as a health centre but it does not fit this description because they offer inpatient services. The government gives them the same amount of money like other health centres. They can not charge the recommended KES 20 because it is not enough to meet the costs of inpatient services. They would have to close the wards..." (Interview, facility in-charge, Kwale)

Concerns about the policy design were raised. Health workers noted that while they wanted to adhere to the 10/20 policy, some elements of the policy were not clear. The exemption criteria were not well understood and in some cases health workers unknowingly charged registration fees for illness conditions and services that ought to be provided for free, as demonstrated by this inaccurate view put forward by one in-charge:

"Before the 10/20 patients with tuberculosis and women attending ante-natal care were not supposed to pay, but with the introduction of the 10/20, everyone has to pay for registration regardless of the type of illness or service received" (Interview, facility in-charge, Makueni)

The practicality of exempting malaria patients from paying the registration fees was also highlighted. Health workers reported that many illness conditions have symptoms similar to malaria, and in the absence of laboratory tests, it is difficult to confirm whether a patient is suffering from malaria. Moreover, the registration fee is paid before the patient is attended to by a health worker, making it impossible for the health worker to exempt the patient from payment should they be diagnosed with malaria:

"The facility still charges registration fees even for malaria patients because it is difficult to differentiate who is suffering from malaria and who is not." (Interview, facility in-charge, Makueni)

Of concern also was = that malaria is reported as the main illness condition in the districts and exempting all malaria patients from paying the registration fees would lead to little or no revenue:

"If it is free [malaria treatment] most people will come to the facility suffering from malaria, all of them will be treated for free and the facility will not raise any money." (Interview, facility in-charge, Kwale)

Lack of clarity partly made health workers-with the support of HFC adjust the fee levels to suit local needs. Even at the district level, the policy was not well understood and it was not always clear what power district officials have over adjusting the official charges. For example, one of the District Medical Officers for Health (DMOH) endorsed new charges formulated by facilities and introduced new district level policies, outlining the maximum acceptable charges for the district.

### Health worker and community perceptions of the 10/20 policy

Health workers and community members reported that the 10/20 policy was a good initiative with the potential to promote access to health care services for all. However, they expressed concerns that limited the potential of the policy to achieve its objectives. For community members, the main concerns regarding the 10/20 policy were low quality of care, drug shortages and long queues:

"Treatment is much cheaper now [post 10/20]. Previously it used to range from KES 200–300 but now we only pay KES 10. But when treatment was expensive [pre-10/20] one would get cured very quickly but now the drugs may not even work...in the past the dispensary had very good drugs... now the treatment is cheap but the drugs do not work [implying they do not always get the appropriate drugs as confirmed by reports from health workers]. So you pay KES 10 and the illness does not go away. It is better to pay more and get cured." (FGD, Men)

Others felt that the drug shortages were not due to the 10/20 policy, but a much deeper problem associated with service provision and staffs remuneration:

"The other day I was told to buy certain drugs for my brother. I set off to go to the chemist but the doctor called me and asked me to give him the money so that he could buy the drugs for me at a cheaper price. Within a few minutes he had bought the drugs....so I wondered whether there was actually a drug shortage or someone is making money out of it." (FGD, men)

"The truth is, the government provides free drugs...but there are a lot of patients and a lot of work and the staffs are poorly paid. So they are forced to sell the drugs to get some money." (FGD, Women)

Health workers expressed similar concerns. Availability of revenue from cost sharing enabled facilities to not only purchase drugs but to also ensure that the kind of drugs available suited local health needs:

"During cost sharing, we had good money to run the facility. We had more drugs because we could buy them. We do not do that any more." (Interview, facility in-charge, Makueni)

Concerns were expressed regarding paying the registration fees and not receiving the prescribed drugs from the facility, with people suggesting that the registration fees should only be paid once the drugs have been received or confirmed to be available from the facility pharmacy:

"Ideally we should not be asked to pay for the prescription book [meaning registration fees]...we should only pay when we receive the actual treatment [meaning drugs]" (FGD, Women)

## Discussion

Adherence to the 10/20 policy was poor in both districts, although facilities in Makueni were more likely to adhere to the policy than those in Kwale. Half the facilities in Makueni and nearly three-quarters in Kwale did not adhere to the 10/20 policy. Of concern was that most facilities in Kwale did not exempt children under five from paying the registration fees. Facility in-charges were aware that under-fives should be exempted from payment but continued to charge them in order to generate additional revenue. An initial evaluation of the 10/20 policy noted that under-fives were exempted from paying the registration fees in almost all facilities [16]. Our findings indicate otherwise, highlighting the limitations of evaluating a policy soon after implementation and the need to monitor adherence over time.

A major factor limiting adherence to the 10/20 policy was drug shortages. Health workers expressed frustrations of not being able to provide patients with appropriate drugs. Drug availability is a major factor influencing health seeking behaviour [19-21]. Severe drug shortages following user fees removal have been reported in other settings [15,22]. In Uganda, where user fees removal was accompanied by other reforms to address the expected increase in utilisation, shortage of drugs and other medical supplies was minimal [23], although information on whether this was sustained is lacking. Reducing user fees may do little to improve access to health care if adequate drug supplies are not maintained and if drug supply does not relate to local health needs [24]. Of concern is that facilities in Kwale reported experiencing regular drug shortages more than in Makueni, despite a new drug supply system (a pull system where facilities order their drugs rather than receiving standard drug kits) being piloted in the district at the time of the study. While we support the move to involve health workers in deciding the contents of the drug kit, we recommend that the pilot system in Kwale be evaluated before a national roll out is implemented to ensure that the benefits of a pull drug supply system are achieved.

One of the main reasons behind the suspension of user fees in Kenya in 1990 was because the concept of charging registration fees was not popular among service users [5,10]. Concerns regarding paying registration fees were also expressed in our study. Whether to charge consultation rather than drug fees remains a critical issue that can undermine trust in the health system in the context of regular drug shortages. The Kenyan health sector should carefully consider whether a flat consultation fee is more appropriate than a flat drug fee to improve access to health care services.

One of the arguments used to support user fees removal in international debates is that they generate very little revenue – usually less than 5% of total operating costs in most countries – and thus removing them will have little impact on revenue and operation of facilities [9,24]. Our findings indicate that although this revenue is low, it serves an important role in ensuring that facilities meet the costs of services and salaries for support staff that are not funded through the government's budget. Various authors highlight the need for government and donors to compensate facilities for lost revenue and for additional resources required to cope with utilisation increases [12,23,24]. In Uganda, where user fees removal was successful – at least in the first year of implementation – the health sector budget was increased by 22 percent to cater for lost revenue, expected drug shortages and increased utilisation. The main aim of the DFF in Kenya was to compensate facilities for lost revenue. However our findings indicate that the DFF did not necessarily lead to improved adherence to the 10/20, with lower adherence found in Makueni, the district with DFF. While health workers acknowledged that the DFF was important in many ways, they expressed concerns about rigid budget lines that did not allow them to reallocate funds between line items, and the fact that the funds could not be used to purchase drugs despite drug shortage being a major problem. Whether fee levels would have been higher in Kwale in the absence of the DFF and whether funds provided under the DFF were adequate to fill the gap in revenue remains unclear. A more detailed assessment of the DFF and why facilities in Kwale may have failed to adhere to the 10/20 policy despite the additional funds is necessary before the nation wide scale up.

Poor policy design, a poor implementation process and staff attitudes towards policy change were major factors that hindered facilities from adhering to the 10/20 policy. Health workers felt that it was practically impossible to implement some elements of the policy and that the exemption criteria were not clear. Consequently, they altered the contents of the policy to suit their settings. Challenges around clarity of the policy content existed in all levels, including the district headquarters. Whether health workers have authority to alter the contents of the 10/20 policy to suit the needs of specific health facilities remains unclear.

Other studies have shown that policy implementers re-design their own policies, influencing the content and impacts of policies particularly in situations where a top-down approach to implementation is adopted [22,25,26]. These authors argue that policy implementers interpret and adopt policy changes in ways that shape policy, leading to unexpected outcomes. Policies may have good intentions, but translating them into practice and ensuring that the intended benefits are achieved can be a challenge [9]. The 10/20 policy demonstrates how the success of a well intended policy can be weakened by poor policy design and implementation. We call for a reduction in the gap between policy makers and implementers by adopting bottom-up approaches to implementation that ensure health workers, facility committees and District Health Management Teams are engaged throughout the policy process.

Affordability of health care services was reported to be better post the 10/20 policy. However, health workers and community members had reservations regarding the perceived quality of care and the potential of the policy to improve access to health care. An initial evaluation of the 10/20 policy indicated that it was popular among patients but not among health workers [16], while our findings indicate that the policy was not popular in either group. Of major concern to the health workers was the high workload, arising from increased utilisation, which exerted pressure on their ability to provide quality services. An initial assessment of the impact of the 10/20 policy reported a dramatic increase in utilisation in the first six months after implementation [16,24]. While increased utilisation may indicate a positive impact of the 10/20 policy, our data suggest that higher utilisation following fees removal should be interpreted with caution. People visited the facility more than once for treatment of the same illness, perhaps because they did not receive appropriate drugs for their condition due to drug shortages. Similar experiences were reported in Madagascar, where utilisation patterns increased following fees removal in 1997/98, but a closer examination of the data revealed that part of the reason for the increase was that many patients were returning for treatment of the same illness due to lack of appropriate drugs or supplies [27].

## Limitations of the study

This study aimed to provide an overview of adherence to the 10/20 policy, 3 years after implementation. A limitation is that it was conducted in only two districts in Kenya and with a small number of facilities purposively located in the poorest parts of the districts. While focusing on a small number of facilities enabled us to collect in-depth information, we recognize the limitations of generalising these findings to other parts of the country. A wide scale evaluation of adherence to the policy that is generalisable to the whole population is necessary. Understanding the impacts of fees removal would have been stronger were data on community and health worker perceptions pre and post 10/20 available. In addition, some of the concerns raised by participants cannot entirely be linked to the10/20 policy, because the public health system suffers from many weaknesses that are beyond the scope of this paper. It is also worth noting that health workers might have lost personal benefits with the introduction of 10/20 (e.g. allowances and salary supplements), which they would have been unwilling to discuss, but which might have influenced their perceptions on the policy. Finally, the results presented are based on people's perceptions which are difficult to verify, hence the findings should be interpreted with caution. Nevertheless important lessons regarding policy implementation and what happens on the ground a few years after implementation can be drawn from the findings.

## Conclusion

In most facilities the 10/20 policy is not being put into practice despite its valuable objectives. We conclude that reduction of user fees in primary health care in Kenya is a policy on paper that is yet to be implemented fully. The findings demonstrate how removing or reducing user fees, though well intended, can have negative implications for service delivery. We join other authors who have called for careful planning before user fees are removed [12,23,24]. We recommend that caution be taken when deciding on how to reduce or abolish user fees and that all potential consequences are considered. Measures to ensure effective implementation of fees reduction or abolition should be put in place through: (1) ensuring that policy guidelines are clearly defined; (2) engaging health workers in the policy design process; (3) providing timely information to health workers, District Health Management Teams (DHMTs) and HFCs; (4) promoting awareness of the policy to community members; (5) providing alternative funds to compensate facilities for lost revenue and to cope with utilisation increases; and (6) monitoring adherence to the policy through for example mystery shoppers and community interviews.

## Competing interests

The authors declare that they have no competing interests.

## Authors' contributions

JC and CM were involved in the conception and design of the study. JM and VO participated in data collection, analysis and writing up. CG supported the design of data collection tools and write up. All authors read and approved the final manuscript.
